# Does the mean adequately represent reading performance? Evidence from a cross-linguistic study

**DOI:** 10.3389/fpsyg.2014.00903

**Published:** 2014-08-19

**Authors:** Chiara V. Marinelli, Joanna K. Horne, Sarah P. McGeown, Pierluigi Zoccolotti, Marialuisa Martelli

**Affiliations:** ^1^IRCCS Fondazione Santa LuciaRome, Italy; ^2^Department of Psychology, University of HullHull, UK; ^3^School of Education, Edinburgh UniversityEdinburgh, UK; ^4^Department of Psychology, Sapienza University of RomeRome, Italy; ^5^Institute of Cognitive Sciences and Technologies, ISTC-CNRRome, Italy

**Keywords:** reading, individual differences, cross-linguistic comparison

## Abstract

Reading models are largely based on the interpretation of average data from normal or impaired readers, mainly drawn from English-speaking individuals. In the present study we evaluated the possible contribution of orthographic consistency in generating individual differences in reading behavior. We compared the reading performance of young adults speaking English (one of the most irregular orthographies) and Italian (a very regular orthography). In the 1st experiment we presented 22 English and 30 Italian readers with 5-letter words using the Rapid Serial Visual Presentation (RSVP) paradigm. In a 2nd experiment, we evaluated a new group of 26 English and 32 Italian proficient readers through the RSVP procedure and lists matched in the two languages for both number of phonemes and letters. The results of the two experiments indicate that English participants read at a similar rate but with much greater individual differences than the Italian participants. In a 3rd experiment, we extended these results to a vocal reaction time (vRT) task, examining the effect of word frequency. An ex-Gaussian distribution analysis revealed differences between languages in the size of the exponential parameter (tau) and in the variance (sigma), but not the mean, of the Gaussian component. Notably, English readers were more variable for both tau and sigma than Italian readers. The pattern of performance in English individuals runs counter to models of performance in timed tasks (Faust et al., 1999; Myerson et al., 2003) which envisage a general relationship between mean performance and variability; indeed, this relationship does not hold in the case of the English participants. The present data highlight the importance of developing reading models that not only capture mean level performance, but also variability across individuals, especially in order to account for cross-linguistic differences in reading behavior.

## Introduction

Reading is a complex task that involves several cognitive and sensory-motor components from image detection to the comprehension of meaning. It takes years to master this skill and during this progression, each of the components undergoes maturation and specific learning effects. Literate adults read with near perfect accuracy at an impressive speed, optimizing each of the processes involved and performing them in parallel. The speeding up of the function may be seen as moving from serial to parallel analysis up to the point in which individuals learn to master orthographic decoding of a letter string in a glance (e.g., Ziegler and Goswami, 2005).

In 1992, Carver proposed a bold conjecture to account for reading rate. Carver showed that readers adjust their reading rate, speeding up if they are searching for a particular word in a text (scanning) and slowing down if they want to memorize concepts. According to Carver, readers may shift “gear” to achieve the desired goal, but they generally read in the middle (third) gear or “rauding” (i.e., reading and auding) which optimizes comprehension considering the speed limits set by the processing components. In a classic paper, Taylor (1965) surveyed the reading skills of 12,000 US students, from first grade to college, and found the average rate to be 300 words per minute (wpm), which was taken by Carver (1992) as an estimate of rauding rate.

This functional measure of reading speed incorporates several components from decoding to motor execution, and it is relatively stable across individuals. Notably, it has been shown that pronunciation time, the most time consuming process, weights heavily on the average speed but contributes minimally to individual differences (Martelli et al., 2014). This means that pronunciation time adds a substantial constant factor to the (much faster) compartment of decoding. Furthermore, it indicates that the maximal reading rate obtained in standard conditions (i.e., rauding) does not necessarily indicate the maximum processing rate for each of the sub-components in reading. Put in different terms, the articulatory component (as well as the eye movement scanning; see below) may pose an upper bound to the estimate of maximal reading rate that can be obtained in functional reading.

In a different line of research, focussed on assessing the perceptual limitations in reading, several authors measured reading speed by means of the Rapid Serial Visual Presentation (RSVP) paradigm. In this procedure, a sequence of words is rapidly presented in the same retinal location. The observer is required to name the words presented (typically a stream of four words per trial) without a time limit. The duration of the words on the screen to achieve a certain level of task performance (typically 80%) is measured. In this paradigm, the articulatory components do not directly exert a role on the estimation of the reading rate, since no time limit is given to complete the response. Furthermore, unlike ordinary reading, the observer does not have to scan for the words to read by eye movements, as stimuli are all presented in the same retinal position. Thus, this procedure minimizes the role of memory, pronunciation time and eye movements, allowing a more direct examination of the decoding components in reading (see Rubin and Turano, 1992; Chung et al., 1998; Legge et al., 2001; Pelli et al., 2007). In fact, compared to other reading techniques, RSVP gives the opportunity to substantially “speed up” reading rate. For example, Potter (1984) originally showed that reading and recall is still excellent at 12 words per second (i.e., 720 wpm), which is much faster than the level of “rauding.”

In the absence of specific reading or visual deficits, and controlling the stimuli for high level cognitive factors, one may assume that decoding is similar across individuals. Indeed, most low-level visual functions, such as acuity or contrast sensitivity, are similar across subjects (Barlow, 1962; Fisher, 1975; Pelli et al., 2006; Strasburger et al., 2011), revealing that perceptual limitations are invariant across individuals and labs. However, when considering the reading speed measurements obtained with RSVP, variability across subjects and labs is, surprisingly, very large. In some cases, the advantage given by the RSVP technique in speeding up reading rate is relatively low, with reading rates around 300 wpm (Latham and Whitaker, 1996; Fine et al., 1997, 1999; Chung et al., 1998; Pelli et al., 2007) while, in other studies, reading rates exceeding 1500 wpm have been reported (Rubin and Turano, 1992; Latham and Whitaker, 1996).

Part of the large discrepancy in RSVP reading across labs may be related to low-level visual effects, such as presence/absence of masking (Felsten and Wasserman, 1980; Breitmeyer, 1984; Enns and Di Lollo, 2000) or to the number of items used in the stream. In particular, in some studies, four words are presented per trial, while in others, number of words well exceeds the memory span (e.g., Latham and Whitaker, 1996; Chung et al., 1998; Yager et al., 1998; Fine et al., 1999; Kwon et al., 2007; Pelli and Tillman, 2007; Pelli et al., 2007; Yu et al., 2007, 2010; Lee et al., 2010; Kwon and Legge, 2012). Note that these studies are mainly concerned with factors affecting visual limitations to reading, such as font size or letter spacing, and much less to cognitive dimensions (as well as to absolute estimates of reading rate which are rarely commented on). Thus, direct comparisons between the various estimates are hard to make since the stimuli are usually not designed to take into consideration linguistic variables (e.g., word frequency, orthographic complexity, orthographic neighborhood, age of acquisition, etc.) that are known to influence speed of reading (e.g., Coltheart et al., 1977; Ferrand and New, 2003).

Furthermore, there is also a surprisingly large discrepancy in reading rate across languages, such as when comparing the irregular English orthography with the consistent Italian one with similar RSVP reading tasks. The reading rate of English 5th and 7th graders with the RSVP of stimuli averages at around 500 wpm (Kwon et al., 2007), while normal 6th grade Italian readers do not exceed 120 wpm, a rate much slower than any other reported for this age level (Martelli et al., 2009). Italian dyslexics' average reading rate is as slow as 40 wpm (Martelli et al., 2009). Although suggestive, comparisons between these two languages are certainly difficult to interpret across experiments, particularly since Italian words tend to be long and morphologically complex, while English words tend to be shorter and morphologically simple.

As described above, most studies on reading focus on group data that average across participants and trials, and only recently it has been suggested that “*it is possible that some of the inconsistencies in the literature may be driven by individual differences among participants*” (Yap et al., 2012, p. 2). The source of this variability may possibly concern strategic differences related to the linguistic demands (both within a language and across different languages). Following Yap et al. (2012), we conjecture that, over and above differences in average speed, variability estimates may also provide insights into the computation involved in reading. Here, we were interested in exploring such variability in relation to differences in orthographic consistency, with the ultimate goal of understanding the invariant and variable properties of reading across languages. Indeed, learning to become a proficient reader in different orthographies may pose very different requirements to the reader and the end product of these different task demands may well be expressed by different degrees of inter-individual variability.

In the present study, we address a number of questions, comparing Italian and English readers. Is there a difference in processing speed of regular and irregular orthographies, once most of the cognitive variables are taken into account? Does the general speed factor interact with the efficiency of the orthographic decoding, as reflected by the size of the lexical effects in the two languages? Indeed, Faust et al. (1999) showed that larger effects of the experimental manipulations are expected in the case of differences in overall processing time across individuals (i.e., larger effects for slower individuals). Do the individual differences across languages arise from different strategies adopted in reading? The difference engine model (DEM), proposed by Myerson et al. (2003), explains group RT differences by assuming that, in the absence of a peripheral deficit, most differences between individuals are due to the amount of cognitive processing required predicting the relationship between mean and SD. Is this relationship as well as vRTs distribution similar across languages in the case of reading tasks? In this study, we attempt to answer these questions through three experiments that compared reading speed (assessed with either the RSVP procedure or with vRT measurements) in a very regular (Italian) and in a very irregular (English) orthography with controlled orthographic materials.

## Experiment 1: processing speed differences between english and italian readers

In this first preliminary experiment, we aim to explore possible differences in processing speed between Italian and English proficient readers, controlling for as many psycholinguistic variables as possible, based on the structural differences between the two languages. Previous observations report large discrepancies in RSVP reading rate across labs and languages, with English observers obtaining much higher estimates of reading rate (e.g., Rubin and Turano, 1992; Latham and Whitaker, 1996; Chung et al., 1998; Kwon et al., 2007; Martelli et al., 2009). However, due to concurrent procedural differences and uncontrolled variables, it is hard to draw a firm conclusion on these data. Here, we test a group of English young adults and a group of peer Italian readers using the RSVP paradigm to confirm the possible presence of different reading rates.

### Materials and methods

#### Participants

Thirty Italian (15 males and 15 females) and 22 English (11 males and 11 females) readers participated in this experiment. Participants were university students recruited from the student population of the Sapienza University of Rome in Italy and of the University of Hull in the United Kingdom. Groups were comparable for age and gender. The age of the Italian group ranged between 19 and 28 years (mean age: 22.96, *SD* = 2.84) with 15.81 (*SD* = 1.39) years of schooling; the age of the English participants ranged between 18 and 24 years (mean age: 20.86, *SD* = 3.77) with 14.23 (*SD* = 1.02) schooling years. All participants were self-reported good readers, without a history of language, reading or spelling disorders. This study, as well as the ones presented in Experiments 2 and 3 (both conducted according to the principles of the Helsinki Declaration) were approved by the Ethical Committee of the Department of Psychology of Rome, and by that of the University of Hull in line with the BPS guidelines. Before taking part in the experiment, the subjects were given a description of the study and approved their participation.

#### Stimuli, apparatus, and procedure

In both languages, words were all nouns, without morphological complexity and irregularity in grapheme-to-phoneme correspondence. Because stress assignment to Italian polysyllabic words is unpredictable by rule, no words with irregular stress were included in the Italian list (i.e., all words were stressed on the syllable before the last). No irregular stress words were used also in the English list. In both languages, archaic, obsolete, poetic and scientific forms were avoided. For the Italian readers, a list of 80 5-letter words were selected from the LEXVAR database (Barca et al., 2002) with frequency ranging from 0 to 100 (mean frequency = 25.1, *SD* = 24.4, Colfis database; Bertinetto et al., 2005). For the English readers, a list of 80 5-letter words was selected from the MRC Psycholinguistic Database 2.0 (Wilson, 1988): Frequency ranged from 0 to 100 (mean frequency: 24.8, *SD* = 36.2 CELEX database, Baayen et al., 1993). Note that, to compare the frequency values of the two databases (the English database has one million of occurrences, while the Italian database counts over three million occurrences), the Italian word frequency values were reported to one million of occurrences. In Appendix A, means (and SDs) of the psycholinguistic variables are reported for the Italian and English lists. The Italian and English lists were matched for frequency, n-size, imageability and age of acquisition (all *ps* > 0.1). Italian and English words were comparable for bigram frequency based on values reported in the MCWord database (Medler and Binder, 2005) for English and in the LEXVAR database (Barca et al., 2002) for Italian language (referring to one million of occurrences). As it can be seen in Appendix A, lists were not matched between languages for number of phonemes [*t*_(159)_ = 7.92, *p* < 0.0001], that was higher for the Italian than the English list. Moreover, it was not possible to match the lists for number of syllables [*t*_(159)_ = 14.41, *p* < 0.0001], as English and Italian differ in the number of syllables and in the complexity of the syllabic structure. The number of syllables is generally higher in Italian (the mode length in the Italian lexicon, according to De Mauro, 1999, is 4 syllables) than in English. Moreover, in English, only 5% of monosyllables are CV (De Cara and Goswami, 2002), while in Italian (as in other romance languages) CV is the most frequent syllable type, covering 56% of syllable tokens in written corpora (for a more detailed description of Italian see Burani et al., 2014; for English, see Wyse and Goswami, 2008).

Words were rendered in Courier New font, a proportionally spaced font, and each letter subtended 0.4° of visual angle. Participants were seated 57 cm away from the computer screen (refresh rate = 60 Hz). A fixation point (a black square subtending 0.2° of visual angle) was presented at the center of the screen for 2000 ms. Immediately after the offset of fixation point, words were presented using the RSVP paradigm, i.e., four words were presented sequentially, one word at a time, at the same location on the display and participants were asked to read them aloud. There was no blank frame (zero inter-stimulus interval) between words. Following Rubin and Turano (1992) no mask was presented prior to the first or after the fourth word in the stream. We measured the duration threshold for each participant by varying exposure duration in a 20-trial run using the improved QUEST staircase procedure with a threshold criterion of 80% correct responses (Watson and Pelli, 1983). The adaptive QUEST procedure increased or decreased the presentation rate (starting from 500 ms) according to the participant's accuracy. Word omissions, mispronunciations and substitutions were considered to be errors. In order for the subjects to familiarize with the RSVP paradigm 10 practice trials (40 4-letter words) were administered prior to the beginning of the experiment. As in the experimental session the word duration in each trial was controlled by the adaptive procedures based on response accuracy.

### Results

**Figure 1:**
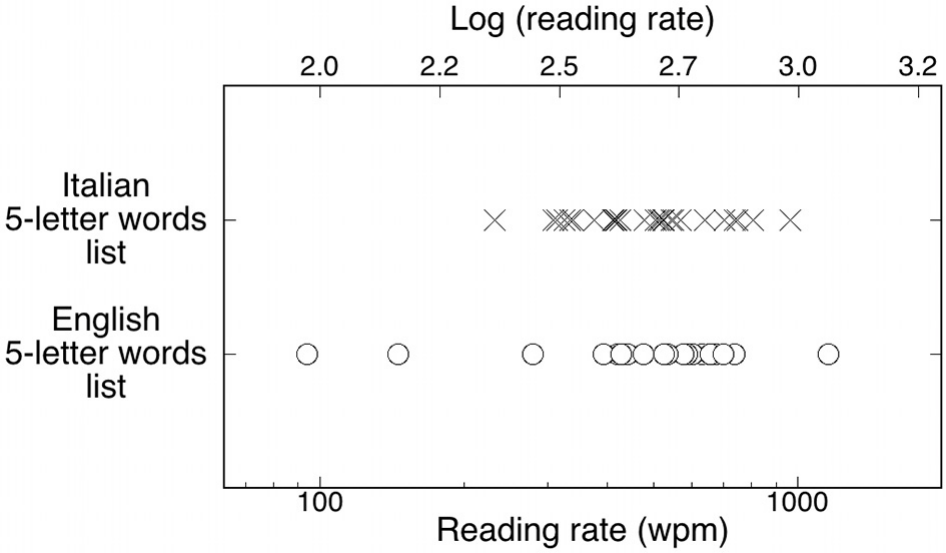
**Individual reading rates for Italian readers (Xs) and English participants (open circles) for a letter-matched list of words**. The upper scale shows the corresponding values expressed as a log (wpm). Note the greater dispersion of experimental points among English than Italian observers.

The reading rate (i.e., wpm) was measured as 60/duration threshold*1000 using the geometric mean as measure of the central tendency of the distribution (represented using a log scale, Figure 1 lower axis) and the 95% confidence intervals (CIs) to express the variability in the distributions. ANOVA comparisons across groups were performed on log-transformed reading rates (linear scale, Figure 1 upper axis). The reading speed for the English list (geomean = 449 wpm; *CI*: 346–583) was not different from the reading speed of the Italian parallel list [479 wpm; *CI*: 433–548; *F*_(1,50)_ < 1, *p* = 0.55]. Results were replicated also when socio-demographic variables (i.e., gender, age, and years of schooling) were added to the analysis as covariates: the main effect of the language factor was not significant [*F*_(1,44)_ about 1; *p* = 0.31]; furthermore, none of the covariates were significant.

Figure 1 presents the reading rate distributions for the Italian and English readers. An inspection of the figure indicates that the English group was less homogeneous than the Italian group, with a larger variability: the group comprised the fastest individual and individuals who were slower (by a factor of ca. two) than the slowest Italian reader. This pattern is confirmed by the Levene's test for equality of variances: the variances of the Italian and English samples were significantly different (*F* = 4.17, *p* < 0.05).

As variability appears as the key feature of the group differences between the two languages we replicated this analysis using untransformed threshold values to check whether the difference in the variance of the two distributions could be due to the adoption of a nonlinear transformation. Mean duration thresholds were 120 ms (*SD* = 44) for the Italian group and 152 ms (*SD* = 142) for the English group. Again, the variances of the two groups were significantly different (*F* = 8.86, *p* < 0.01). Therefore, it appears that the difference in variability between the two languages is not due to the use of a nonlinear transformation of data.

#### Comments

Contrary to expectations based on our preliminary observations, and the work of Paulesu et al. (2000), Italian readers as a group were neither faster nor slower than English readers once the items were made comparable for some relevant psycholinguistic variables. However, the two groups showed substantial differences in individual variability with the Italian group considerably more homogeneous than the English one. The English group included both the fastest participant, reading over 1100 wpm, and the slowest participant, reading at ca. 90 wpm. Clearly, this phenomenon is captured by the variability in the two distributions and not by the group mean. This large variability is somewhat coherent with the 5 to 1 difference across labs testing English RSVP reading (Rubin and Turano, 1992; Latham and Whitaker, 1996; Fine et al., 1997, 1999; Chung et al., 1998; Pelli et al., 2007).

If high individual variability is the norm, the mean performance of any given sample would depend upon the actual proportion of fast and slow individuals. This is particularly the case for RSVP studies which are typically concerned with perceptual parameters and use a large number of trials but a small (often very small) sample size. In these conditions, variability between samples is expected to be quite high and this may substantially contribute to the very different reading rates reported in the literature.

Variability may be the diagnostic marker of the reading differences across regular and irregular orthographies. However, this first preliminary experiment had several pitfalls preventing any definite conclusion on whether the high inter-individual variability among English observers is a “real” phenomenon. Obviously, one possible source of variability would be the presence of a proportion of individuals with a reading deficit. All participants were self-reported proficient readers, but, given the absence of an independent evaluation using standardized reading measures, it is impossible to exclude such an explanation with certainty. Moreover, we did not have a measure of wpm in the case of words equated on number of phonemes (rather than letters). Based on these considerations it seemed important to confirm and extend the findings of Experiment 1 with a new group of subjects; this was carried out in Experiment 2. Additionally, it is unclear whether the difference in variability between the two groups is specifically related to the cognitive components involved in the performance with the RSVP or may be a more general phenomenon extending across reading tasks. This was the aim of Experiment 3.

## Experiment 2: functional reading abilities and RSVP reading speed

In this experiment we aimed to replicate Experiment 1 measuring RSVP reading speed in an independent sample. In order to exclude differences between samples related to more general cognitive efficiency and/or the presence of a reading deficit, standardized tests appropriate for the participants' age and language were administered to ensure that all participants were normal fluent readers. Additionally, the performance of English and Italian readers was examined both using lists of words matched for number of letters and lists of words matched for number of phonemes.

### Methods

#### Participants

Italian readers were 32 university students recruited from the student population of the Sapienza University of Rome; the English participants were 26 students recruited from the student population of the University of Hull. As shown in Table 1, the groups were matched for age (*t* < 1; *p* = 0.59); the years of schooling were higher [*t*_(56)_ = 9.11, *p* < 0.0001] for the Italian than the English sample. These differences are presumably related to the longer Italian schooling system.

**Table 1:** **Socio-demographic information and reading and Raven's SPM performance for the Italian and English samples of Experiments 2 and 3**.

	**Italian participants**	**English participants**	**Difference**
Gender	15M, 17F	11M, 15F	X^2^ < 1, p = 0.73
Mean age	23.8 (1.9)	23.0 (1.1)	T < 1, p = 0.59
Years of schooling	17.1 (1.6)	14.6 (0.5)	t_(56)_ = 9.11, p < 0.0001
Raven's SPM (mean standard score)	110 (9.6) (range: 93–128)	108 (10.4) (range: 90–140)	T < 1, *p* = 0.82
Word reading: errors (mean z score)	−0.24 (0.67)		
Word reading: speed -syllables/second- (mean z score)	−0.49 (1.03)		
Pseudo-word: reading errors (mean z score)	−0.38 (0.66)		
Pseudo-word: reading speed -syllables/second- (mean z score)	−0.11 (0.80)		
Word reading: TOWRE sight word efficiency (mean z score)		0.18 (0.54)	
Pseudo-word reading: TOWRE Phonemic Decoding Efficiency (mean z score)		0.65 (0.65)	

*Unless otherwise specified, values in brackets indicate standard deviations*.

The following inclusion criteria were used to select the participants included in the two samples (English and Italian): (i) absence of neuro-sensory deficits or cognitive impairment (as assessed by Raven's Standard Progressive Matrices—SPM, Raven, 2008). (ii) Absence of a reading deficit assessed by single word and pseudo-word reading tests (for Italian: Martino et al., 2011; for English: the Test of Word Reading Efficiency—TOWRE, Torgesen et al., 1999); (iii) Normal or corrected to normal visual acuity; (iv) absence of a history of reading disorder. Table 1 reports the performance obtained by the two language groups in the standard reading tests and the Raven's SPM.

#### Stimuli, apparatus, and procedure

Two lists of 80 stimuli were generated for each language, one consisting of words of 5 letters and one consisting of words of 5 phonemes. Again, words were all nouns: morphologically complex, archaic, obsolete, poetic and scientific words as well words with an opaque grapheme-to-phoneme correspondence or irregular stress were avoided in both languages. Words were selected from the MRC Psycholinguistic Database 2.0 (Wilson, 1988) for English and from the LEXVAR (Barca et al., 2002) and Colfis (Bertinetto et al., 2005) databases for Italian language. Note that for words selected by the Colfis database, values of n-size, imageability, age of acquisition and bigram frequency are computed with the same procedure used for the LEXVAR database (Barca et al., 2002). Table 2 reports the values of frequency, number of letters and phonemes for each list. Note that for the English readers, the 5-letter list was the same as that used in Experiment 1.

**Table 2:** **Characteristics of Italian and English 5-letter and 5-phoneme words in Experiment 2**.

		**Italian**	**English**
5-Letter words	No of phonemes	4.15 (0.36)	4.01 (0.70)
	Word frequency (mean)	29 (64)	25 (36)
5-Phoneme words	No of letters	5.45 (0.69)	6.03 (0.90)
	Word frequency (mean)	27 (88)	22 (24)

*Note that the word frequency values were computed for Italian according to the Colfis database (Bertinetto et al., 2005; based on one million occurrences) and for English according to the CELEX database (Baayen et al., 1993). Values in brackets indicate standard deviations*.

The Italian and English lists were matched for frequency, n-size, imageability and age of acquisition (all *ps* > 0.1) but not bigram frequency (with a higher value of bigram frequency in the Italian relative to the English lists, according to MCWord database in English, and LEXVAR database in Italian; for the letter-matched list: *t*_(159)_ = 4.63, *p* < 0.0001; phoneme-matched list: *t*_(159)_ = 5.20, *p* < 0.0001. Also, lists were not matched for number of syllables since Italian words generally have higher values than English words [for the letter-matched list: *t*_(159)_ = 14.41, *p* < 0.0001; phoneme-matched list: *t*_(159)_ = 4.96, *p* < 0.0001]. Appendix B reports the means (and standard deviations) of the psycholinguistic variables for each experimental list.

The apparatus and procedure were the same as in Experiment 1.

### Results

Differences between the two language groups on the log transformed reading rates were assessed through an ANOVA with language (English, Italian) as the unrepeated factor and list (letter-matched, phoneme-matched) as the repeated factor. The results indicated a significant main effect of the language factor [*F*_(1, 56)_ = 13.46; *p* < 0.001] with the English readers faster than the Italian ones on both the letter-matched list (geomean = 453 and *CI*: 344–598 for the English readers; 325 wpm and CI: 292–362 for the Italian readers) and the phoneme-matched list (geomean = 514 and *CI*: 407–649 for the English readers; 299 wpm and *CI*: 266–337 for the Italian readers). The main effect of list [*F*_(1,56)_ < 1, *p* = 0.68] and the language by list interaction [*F*_(1, 56)_ = 2.7; *p* = 0.12] were not significant. Results were replicated also when socio-demographic variables (i.e., gender, age, scholarity and Raven's SPM accuracy) were added to the analysis as covariates: only the main effect of the language factor was significant [*F*_(1, 55)_ = 9.78; *p* < 0.01]; no other main effect or interaction were significant, as well as the effect of the covariate variables.

**Figure 2:**
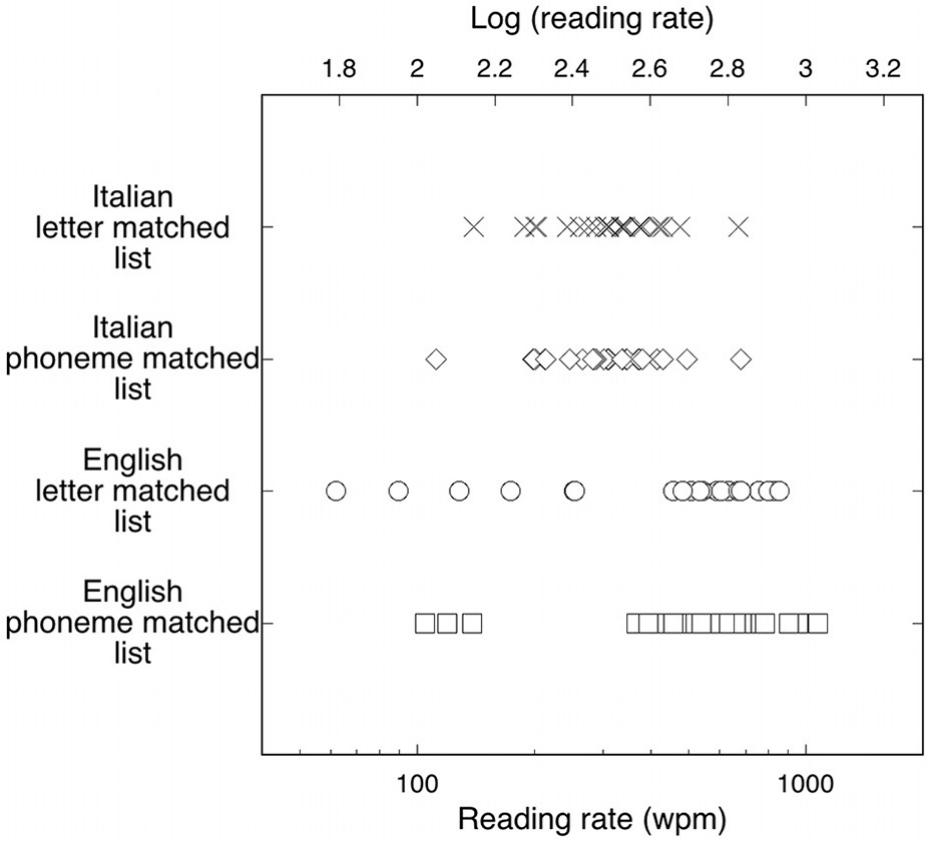
**Individual reading rates for Italian readers (Xs and diamonds for letter- and phoneme-matched lists, respectively) and English subjects (open circles and open squares for letter- and phoneme-matched lists, respectively)**. The upper scale shows the corresponding values expressed as a log (wpm). Reading rates for English observers are much more variable than rates for Italian observers.

Figure 2 shows the reading rates obtained by each individual for the two lists. As in Experiment 1, an inspection of the figure reveals much greater variability in the English than in the Italian sample. This is confirmed by the Levene's test for equality of variances (for the 5-letter list: *F* = 12.20, *p* < 0.001; for the 5-phoneme list: *F* = 4.40, *p* < 0.05). The English group contains both the fastest individual (reading at 1075 wpm) and the slowest individual (reading at 62 wpm).

Again, to control for the possible effect of introducing a nonlinear transformation on the difference in the variance of the two distributions, we made the same analysis in terms of untransformed threshold values. For the 5-letter list, mean duration thresholds were 112 ms (*SD* = 38) for the Italian group and 175 ms (*SD* = 213) for the English group. For the 5-phoneme list, duration thresholds were 123 ms (*SD* = 48) for the Italian group and 137 ms (*SD* = 135) for the English group. The Levene's test for equality of variances indicated that the variances of the two groups were different for the 5-letter list (*F* = 15.88, *p* < 0.0001) as well as the 5-phoneme list (*F* = 6.94, *p* < 0.01). These results indicate that the differences in variability between the two languages are genuine, i.e., they are not due to the use of a nonlinear transformation of data.

#### Comments

Unlike Experiment 1, but in agreement with our informal observations based on children's data, the results revealed that English readers were on average faster than Italian readers. However, the results also replicated the much greater variability in the English sample compared to the Italian one, already observed in Experiment 1. In this experiment, we directly evaluated the subjects' reading proficiency in standard reading tests. Therefore, the large asymmetry in reading rates was present even after controlling for reading proficiency, indicating that differences in variability across languages may be a true phenomenon that needs to be explained.

Differences in average rate, absent in Experiment 1 and present in Experiment 2, may be interpreted, at least in part, as due to the sampling effect from a greatly variable distribution. Thus, as English individuals are likely to show extreme performance on both ends of the distribution, the relative proportion of such “fast” and “slow” individuals may greatly influence the general outcome of a study, unless a very large sample is examined. Note that in psychophysical studies using RSVP of stimuli, sample sizes are usually quite small and several experiments are actually run on very experienced observers (including experimenters).

One note of caution should be advanced in relation to the proficiency measures used in the two languages. We relied on standardized, validated procedures widely used in the two linguistic contexts. Clearly, it was not possible to use fully comparable instruments as different tasks and measures are traditionally used in the two clinical settings. Notably, English observers were all considered normal at a standard reading test which included an evaluation of speed; the TOWRE (Torgesen et al., 1999) uses a combined measure of speed and accuracy, based on the number of words (or pseudo-words) accurately read within 45 s. In this respect, it should be kept in mind that, in clinical testing, performance is measured with reference to typical samples, usually in terms of the standardized distance from the mean. So, large variability in the data would allow for greater distances from the mean to be accepted as normal. In other terms, there is no absolute way to establish normality other than in comparison to a group of individuals without apparent reading difficulties. So, if variability in reading speed is the norm, it will prove relatively difficult to be considered “atypical” in this particular measure. Finally, it should be kept in mind that, by limiting the influence of articulatory and eye movement components, the RSVP procedure allows for a much larger spread of measure than standard reading (which finds its upper limit with “rauding”; Carver, 1992).

In keeping with this last observation, we wondered whether the large variability in rates shown by readers of languages with irregular orthographies both here and in the literature is related to the characteristics of the RSVP paradigm or extends to other reading measurements. One widely used measure of reading speed is vocal RTs. They are usually measured to address the effect of lexical variables and build models that aim to explain reading. For example, the recent Connectionist Dual Process model (CDP++ model, Perry et al., 2010) simulates the effects found in reading aloud mono- and di-syllabic words and pseudowords, in stress assignment, regularity and syllable number. Furthermore, there is a large literature concerning the interpretation of RT measurements and, in particular, there are models developed to account for individual differences in this measure. According to Wagenmakers and Brown (2007), the three general characteristics of RT distributions that need to be accommodated by any model are that: (a) RT distributions are typically skewed to the right; (b) this skew increases with test difficulty; and (c) the spread of the distribution grows as a function of the mean. Indeed, various models have been developed to tackle this last question, i.e., to account for the relationship between the mean and the standard deviation of a response time distribution (e.g., Faust et al., 1999; Myerson et al., 2003), and they can be particularly fruitful in the present context. Thus, with the aim of addressing the selectivity of the variability effect across languages, in Experiment 3 we extended our observations to vRT measurements.

## Experiment 3: examining the mean and the standard deviation

Despite the emphasis given by most models of reading on the prototypical reader, there is clear evidence that variation in reading skills uncover the underlying process of reading (Balota and Spieler, 1999). To date, the systematic study of individual differences in RT measures has been particularly focussed on aging (Salthouse, 1985; Cerella, 1990) and practice (e.g., Logan, 1992) effects and much less so on understanding the performance of young proficient adults.

One line of investigation has focussed on the possible modulating role of general speed of processing differences across groups. It has been noted that, to fully investigate selective effects of experimental manipulations (e.g., frequency effect), the global factor influencing the differential speed of processing across groups must be taken into account (Faust et al., 1999). Studies have found that more difficult conditions (e.g., low frequency long words compared to high frequency short words) produce larger differences in generally slower groups of individuals (e.g., older adults) due to over-additive interactions (Salthouse, 1985; Cerella, 1990; Myerson et al., 1992; Faust et al., 1999). In line with the presence of an over-additivity effect, Paulesu et al. (2000) reported a larger lexicality effect (words read faster than pseudo-words) in the generally slower sample of English readers than in their sample of Italian readers (who were faster readers). In a developmental perspective, Zoccolotti et al. (2009) found a lexicality effect from grade 1 to grade 8; however, the effect increased progressively with age, when the role of over- additivity was controlled for. In this vein, we will compare vRTs as a function of task difficulty (manipulating a variable such as word frequency) between two languages that, as we have seen in Experiments 1 and 2, differ in terms of mean performance (as well as in variability). One aim of the experiment was to assess the relationship between individual differences between groups and the role played by a lexical variable (i.e., word frequency).

A second line of investigation focussed on the characteristics of the distribution of vRTs. There is a large literature that examined which is the most appropriate distribution to describe the typical skew observed with RTs. In this vein, possible candidates are the ex-Gaussian, the shifted lognormal, the shifted Wald, the shifted Weibull, and the Gumbel distribution (for a discussion among these options see Wagenmakers and Brown, 2007). Yap et al. (2012) extensively investigated individual differences in the reading performance of young English adults in relation to vocabulary knowledge by applying the ex-Gaussian analysis (i.e., a convolution of a Gaussian and exponential distribution) to investigate the RT distributions in reading (Ratcliff, 1979). Interestingly, individual differences were associated with diverse distributional patterns and cognitive abilities (Yap et al., 2012). In particular, results emphasized the role of stable lexical processing characteristics at the individual level. Interestingly, different ex-Gaussian parameters were differentially sensitive to lexical knowledge; thus, the correlation between vocabulary knowledge and vRTs was greatest for the parameter (τ) expressing the exponential component (particularly sensitive to the tail of the RT distribution). Following these observations, we will apply the ex-Gaussian analysis to investigate the RT distributions and the modulating role of a lexical variable such as word frequency in reading of English and Italian proficient readers.

Finally, an interesting line of research on RTs is the development of general models that try to understand the individual performance by decomposing this measure into its constituents. For example, in explaining the relationship between task difficulty (expressed as average speed) and individual differences (measured by SDs), Myerson et al. (2003) proposed the DEM, a two-compartment model. Accordingly, the observers' response is related to a sensory-motor compartment that is generally invariant across subjects (including fast and slow populations, such as old and young adults), and a cognitive compartment that determines how individual differences vary as a function of task difficulty. Critically, the DEM envisages specific predictions to evaluate the relative contributions of the two compartments. These predictions will be tested in the present sample of English and Italian readers.

The general aim of the experiment was to extend the RSVP results to the vRT measures and to assess individual differences within and between groups and the role played by a lexical variable (i.e., word frequency). In examining these questions we took advantage of the previous general literature on RT measures. Thus, we examined (a) the possible presence of over-additivity effects; (b) the distribution of vRTs by ex-Gaussian analysis; and (c) the fit of vRT measures to the DEM (Myerson et al., 2003).

### Methods

#### Participants

The same participants in Experiment 2 also took part in Experiment 3.

#### Stimuli, apparatus, and procedure

Stimuli used for the vRTs experiment were selected from the two lists of 80 words used in each language for the RSVP experiments. A sub-list of 20 high-frequency words and one of 20 low-frequency words was created from both the 5-letter and 5-phoneme lists (see Table 3 for a description of the lists).

**Table 3:** **Characteristics of Italian and English 5-letter and 5-phoneme (high- and low-frequency) words in Experiment 3**.

			**Italian**	**English**
5-Letter words	Low frequency words	No of phonemes	4.25 (0.44)	4.00 (0.76)
		Word frequency (mean)	3 (1)	3 (2)
	High frequency words	No of phonemes	4.05 (0.22)	4.27 (0.80)
		Word frequency (mean)	57 (57)	61 (19)
5-Phoneme words	Low frequency words	No of letters	5.70 (0.69)	6.33 (1.05)
		Word frequency (mean)	3 (4)	3 (1)
	High frequency words	No of letters	6.00 (0.82)	6.00 (0.85)
		Word frequency (mean)	55 (79)	64 (15)

*Note that words frequency values were computed for Italian according to the Colfis database (Bertinetto et al., 2005; based on one million occurrences) and for English according to the CELEX database (Baayen et al., 1993). Values in brackets indicate standard deviations*.

In Italian and English (for both the 5-letter and 5-phoneme stimuli), the sub-lists of high- and low-frequency words did not differ for imageability, number of letters, phonemes, N-size and bigram frequency (all *ps* > 0.1), but differed for age of acquisition (as expected due to the high correlation with frequency). The four sub-lists (5-phoneme high-frequency words, 5-phoneme low-frequency words, 5-letter high-frequency words, and 5-letter low-frequency words) in English did not differ from the Italian sub-lists for any variable considered, except for the number of syllables, which were higher in Italian than in English for the 5-letter sub-lists. The means (and standard deviations) of these psycholinguistic variables are reported for each set of experimental stimuli in Appendix C.

Participants were seated ca. 57 cm from the computer screen. Stimuli were presented using the E-prime 2 software. Each trial began with a fixation point that remained on the screen for 500 ms. Subsequently, a word appeared in the same position. The stimulus remained on the screen until the participant responded. High and low frequency words were randomized for each participant and presented in separate blocks. The order of presentation of the two blocks was balanced across subjects. Five practice stimuli preceded each block. The participant was requested to read the stimulus as quickly and accurately as possible. VRTs were recorded using a voice key (S-R Box). The computer recorded the onset of the vocal response. The experimenter manually recorded pronunciation errors. The responses were tape-recorded to allow offline rechecking. The vRTs corresponding to errors were excluded from the analyses. Self-corrections and wavers were considered errors and the corresponding vRTs were not included in the analyses. Invalid responses (due to technical problems) and vRTs below 200 ms were also excluded from the analyses (1.8% in the English sample and 2.0% in the Italian sample).

### Results

#### Frequency effect as a function of language

Table 4 reports the means (and standard deviations) of vRTs and error rates of Italian and English participants in each condition of the experiment. As it can be seen from the table, the percentage of errors was very low for both groups and, so, no formal analysis of error measures was made. The results on vRTs were submitted to an ANOVA with language as the unrepeated factor, and list (letter- and phoneme-match) and frequency (high and low) as repeated measures.

**Table 4:** **Means (and standard deviations) of vRTs and error rates (% of errors) of Italian and English participants for all experimental conditions of Experiment 3**.

**Words**		**vRTs (ms)**				**Errors (%)**			
		**Italian participants**		**English participants**		**Italian participants**		**English participants**	
		**Mean**	***SD***	**Mean**	***SD***	**Mean**	***SD***	**Mean**	***SD***
5-Phonemes	High frequency	494.7	51.5	474.4	62.1	0.9	0.3	0.8	0.4
5-Phonemes	Low frequency	514.4	65.6	507.4	82.3	0.5	0.3	0.4	0.3
5-Letters	High frequency	488.0	61.3	476.4	63.9	1.3	0.4	0.6	0.4
5-Letters	Low frequency	522.7	71.1	505.8	79.0	0.6	0.3	0.2	0.3

The main effect of frequency was significant [*F*_(1, 56)_ = 86.59; *p* < 0.0001]: low-frequency words were read slower (511 ms) than high-frequency words (482 ms). No other main effects or interactions were found to be significant: vRTs of the two groups did not differ (English observers: 491 ms, Italian observers: 502 ms; *F* < 1, *p* = 0.50), and the two lists were equivalent in terms of reading speed (letter-match: 496 ms, phoneme-match: 497 ms; *F* < 1, *p* = 0.95). Thus, in the absence of a general speed difference between the two linguistic groups, the effect of word frequency was present but did not vary between the two language groups. Results were replicated also with socio-demographic variables (i.e., gender, age, years of schooling and Raven's SPM accuracy) added to the analysis as covariates: only the main effect of frequency was significant [*F*_(1, 55)_ = 9.32; *p* < 0.01]; no other main effect or interaction was significant, as well as no effect of the covariate variables.

#### Individual difference distribution as a function of language

We characterized the vRT distributions of English and Italian participants in terms of the ex-Gaussian probability density functions. The ex-Gaussian distribution is the convolution of a Gaussian (normal) and exponential distribution that accounts for the positively skewed RT distribution often seen in empirical data. We used the MatLab analysis tools provided by Lacouture and Cousineau (2008) and applied the following ex-Gaussian function:

(1) $$f(x|\mu ,\sigma ,\tau )=\frac{1}{\tau }\exp\left(\frac{\mu }{\tau }+\frac{\sigma^{2}}{2\tau^{2}}-\frac{x}{\tau }\right)\Phi \left(\frac{\text{x-}\mu -\frac{\sigma^{2}}{\tau }}{\sigma }\right)$$

in which the exponential component (exp) is multiplied by the cumulative Gaussian component (Φ). The resulting ex-Gaussian distribution contains three parameters: mu (μ) and sigma (σ) are the mean and standard deviation of the Gaussian distribution, and tau (τ) is the mean of the exponential component. We estimated the three parameters values of the individual participants' data distributions across items by applying the maximum likelihood procedures described by Lacouture and Cousineau (2008). Appendix D presents the individual mean vRTs, its standard deviation, as well as the ex-Gaussian parameters for individual participants across all conditions (letter- and phoneme-matched, high- and low-frequency lists).

In keeping with the analyses carried out in Experiments 1 and 2, we examined the various ex-Gaussian parameters both in terms of group differences (by means of *t*-tests) and in terms of equality of variances (by means of Levene's test). Means (and SDs) and results of these analyses are presented in Table 5.

**Table 5:** **Means (and standard deviations) for the ex-Gaussian parameters of Italian and English participants across experimental conditions of Experiment 3**.

**Ex-Gaussian parameters**	**Italian participants**		**English participants**		**Student test**		**Levene test**	
	**Mean**	***SD***	**Mean**	***SD***	***t***	***p***	***F***	***p***
Mu	439.0	45.9	445.6	59.7	−0.46	0.64	0.36	0.55
Sigma	35.7	18.7	75.9	32.3	−5.63	<0.0001	8.67	0.005
Tau	66.7	28.0	45.3	40.3	2.30	0.026	7.42	0.009

*Group comparisons (by Student t-test) and Levene's test for equality of variances are presented*.

*T*-test comparisons revealed a significant difference between the standard deviation (σ) of the Gaussian component but no difference in the mean: Italian observers showed a μ of 439 ms and a σ of 36, while English observers a μ of 446 ms and a σ of 76. Furthermore, the τ (representing the mean of the exponential component) was significantly larger in the Italian (67 ms) than in the English (45 ms) group.

The Levene's test for equality of variances indicated that the variances of the two groups were not different in the case of the μ parameter while they were significantly different for the σ and τ parameters, in both cases indicating greater individual variability in the English than in the Italian sample (see Table 5).

To summarize these results: (a) the two linguistic groups were similar in μ both in terms of mean performance and inter-individual variability; (b) Italian observers showed higher τ and lower σ values than English observers; and (c) independent of group mean differences, English observers were more variable across individuals for both τ and σ, but not μ.

**Figure 3:**
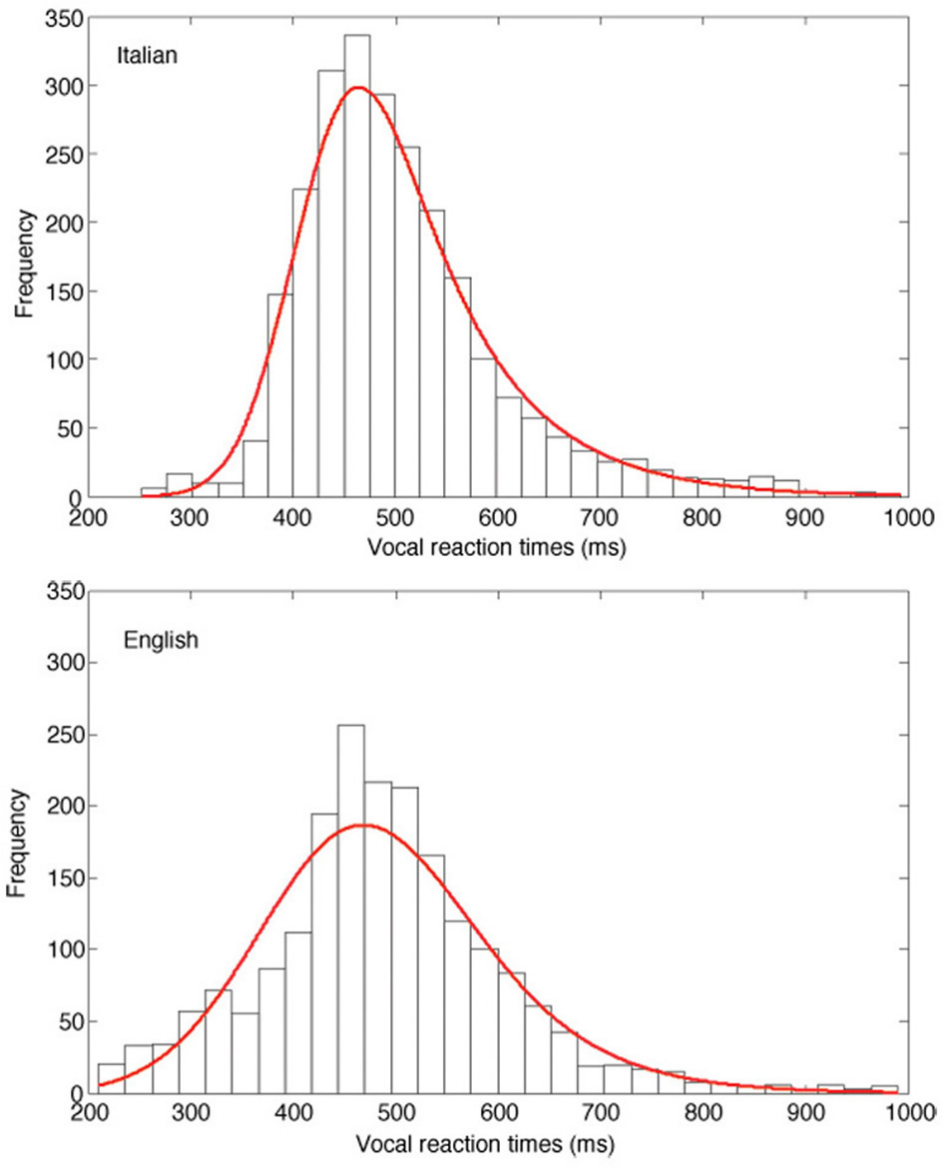
**Frequency distribution of vRTs of the Italian (upper panel) and the English (lower panel) sample**. The red solid lines represent the best fit of the ex-Gaussian distributions of the data obtained by the two samples.

Figure 3 presents the fits of the ex-Gaussian functions across participants to the empirical data separately for the two linguistic groups, using a super-subject approach (as in Balota and Spieler, 1999). In agreement with the individual data, the results of the fit of the Italian data resulted in a μ of 416 ms, a σ of 49, and a τ of 89, while the English fit indicated a μ of 412 ms, a σ of 89, and a τ of 78. As shown by the figure, the larger τ obtained by Italian readers is evident in the positively skewed vRT distribution (upper panel) relative to the English data distribution (lower panel), while a larger variability characterizes this latter group.

In order to clarify the relation between the distributional parameters and the lexical status of the stimuli we applied the ex-Gaussian fit separately for the two lists of high and low frequency words. It must be noted that due to the limited number of items (40 for each participant) parameter estimates may only be taken as a suggestion of an existing relationship. Three separate ANOVAs were applied to the resulting parameters with group (English and Italian) as unrepeated factor and frequency (high and low) as repeated factor. The results for the τ estimates revealed the significant main effect of group and frequency [respectively: *F*_(1, 56)_ = 8.04, *p* < 0.01; *F*_(1, 56)_ = 22.55, *p* < 0.0001] but not of their interaction (*F* < 1, *p* = 0.79). The relative means for the τ parameter were 40 for the English group and 65 for the Italian, and 41 for high- and 67 for low-frequency words. As for the σ parameter only the main effect of group was significant [*F*_(1, 56)_ = 37.26, *p* < 0.0001], with a larger value for the English sample (72.5) relative to the Italian (34.5). The main effect of frequency and its interaction with group were not significant (*F* < 1, *p* = 0.87 and *F* < 1, *p* = 0.77, respectively). No main effect (group: *F* < 1, *p* = 0.53; frequency: *F* < 1, *p* = 0.63) or interaction was significant in the μ component of the distributions (*F* < 1, *p* = 0.97).

The results indicate that the τ (but not the μ and σ) parameter is sensitive to the effect of frequency. As in the general analyses presented above, larger τ values were present for Italian individuals but no differential effect of language on the frequency effect was detected.

#### Modeling vRTs as a function of language

In order to investigate the nature of the individual differences between the two groups we applied the DEM (Myerson et al., 2003). According to this model, the slope of the linear relationship between mean vRTs and SDs is indicative of the amount of processing required by the observers to perform the task and it directly assesses the cognitive compartment (i.e., the slope indicates the correlation between the cognitive stages involved in the task). By contrast, the intercept on the x-axis of this linear relationship estimates the time of the non-decisional sensory-motor compartment (which is supposed to be invariant across observers). Thus, the DEM allows for independent estimations of the cognitive and sensory-motor components in determining individual differences in task performance.

**Figure 4:**
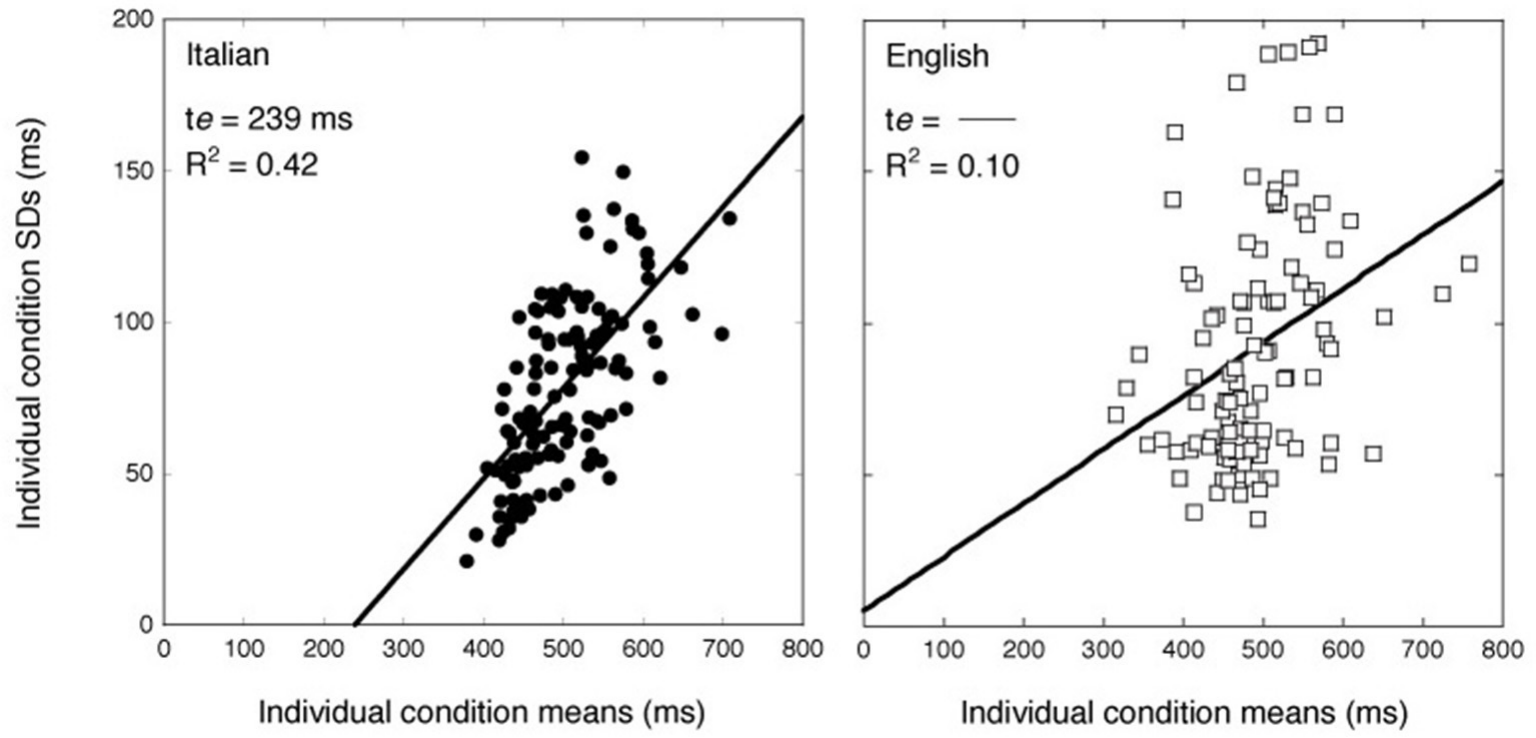
**Individual participants' SDs over items as a function of mean vRTs**. On the left Italian readers; on the right, English participants. The solid line represents the best fit of the DEM to the data (Myerson et al., 2003).

Figure 4 shows the relationship between individual SDs and vRTs in the two groups: data for Italian observers are presented on the left, while those of English observers are shown on the right. The variability grows linearly with increasing condition means for Italian readers; this is less clear for English readers. The solid line in Figure 4 represents the DEM prediction calculated on all the participants using the following equation (Equation 2):

(2) $$SD=\left(r-\frac{\sigma_{c}}{\alpha }\right)\left(RT-t_{e}\right)$$

where σ, α, *t_e_*, and *r* are parameters that are free to vary and represent the variance and amplitude of the effects, the time required by the sensory-motor compartment, and the theoretical correlation between the cognitive stages, respectively. In Figure 4, the sensory-motor compartment is represented by the x-intercept of the regression line. In the case of the Italian sample, the model explains relatively well the variability in the data (*R*^2^ = 0.42) with an estimated time for the sensory-motor compartment of 239 ms and a slope of 0.30. Note that these values are close to those typically reported in the literature (Myerson et al., 2003). By contrast, the model does not account well for the English data (*R*^2^ = 0.10). The slope relating means and SDs is nearly flat (0.17) and no reliable estimation of the sensory-motor compartment is possible; indeed, the x-intercept of the regression line is negative (−26 ms).

#### RSVP and vRT reading comparison

As the same subjects participated in Experiments 2 and 3 this allowed examining the consistency of individual differences between the RSVP (in terms of the log of wpm), and the vRT measures. The Pearson correlation between the two measures was 0.35 for the Italian participants (*p* < 0.01) and 0.31 for English participants (*p* < 0.05). The reading measures in Experiments 2 and 3 differ in terms of absolute performance level and of the response compartment involved. RSVP reading thresholds are calculated at a criterion level of task performance of 80%, while vRTS are measured for correct responses (ideally 100% correct). In addition, while RSVP maximizes the decoding component of the process leaving unlimited time to utter the word, vRT measures include the programming and the beginning of the motor execution (for the role of motor compartment on vRT and total time measures see Martelli et al., 2014). Nonetheless, the analysis shows that the two measures are significantly correlated.

#### Comments

Do the reading skills measured by the speed in vRTs interact with the size of the lexical effects? Estimation of reading skills did not reveal a mean group difference between a regular and an irregular orthography in adult proficient readers. In this context, standard analyses based on mean performances did not show a significant interaction between frequency and language, indicating a similar use of the stimulus lexical properties by the two groups.

The analysis of the ex-Gaussian probability density functions revealed that language differences are captured by the differential weight of the two components (Gaussian and exponential) in determining the vRT distributions of the two linguistic groups: Italian observers showed higher τ and lower σ values than English observers while no group difference was detected in the case of μ values. As a consequence, examining differences across regular and irregular orthographies only with reference to the mean fails in capturing the phenomenon. Additionally, the results indicate that τ, but not σ and μ, are modulated by the lexical status of the stimuli. These findings are in keeping with previous data from Yap et al. (2012) who reported that vocabulary knowledge was correlated to τ more highly than to μ in speeded pronunciation of words (as well as in a lexical decision task). More generally, similar results have also been reported for decision and selective attention tasks (Schmiedek et al., 2007; Tse et al., 2010).

A critical interest of the present study is to examine the possible presence of inter-individual differences between the two linguistic groups. Results indicated a profile of individual differences that varied as a function of the ex-Gaussian parameters: English observers were more variable for τ and σ but not for μ. In comparing data from this experiment to the findings of Experiments 1 and 2 note that in the case of RSVP paradigm only mean performance values are available. So, in this vein, one should note the differential outcome with the two paradigms: in the case of the RSVP, one obtains a different spread of performances with the English sample containing the fastest and slowest individuals. In the case of vRTs, this differential spread is not present (as indicated by the pattern of data in the case of μ values). However, English observers were more variable both in terms of σ and τ. The former finding indicates greater intra-individual variability (further comments will be advanced in the general discussion). Of particular interest is the greater variability in τ as this parameter is the one that selectively captures the effect of the lexical status of the stimuli.

One of the most basic results in the RT literature is that slower vRTs are accompanied by higher variability (Wagenmakers and Brown, 2007). This is commonly true of both group and condition comparisons. So, older people are generally slower and more variable than younger individuals; more difficult conditions are invariably associated with larger variability values (Faust et al., 1999; Myerson et al., 2003). By contrast, in the current study, English participants were more variable but not slower than Italian participants across conditions. So, the pattern shown by English readers is at odds with the basic predictions of models interpreting global effects of performance. Note that here we are showing individual observer's means and SDs separately for each condition. Myerson et al. (2003) DEM is typically applied to condition means segregating slow vs. fast subjects (e.g., comparing the fast to the slow quartile of the observers distributions) in studies comprised of several independent measures. Application of DEM indicates that fast and slow subjects are described by the same relationship between means and SDs. The consequence of these linear relations is that the difference between the vRTs of the subgroups of fast and slow processors increases proportionally with the average vRT as SDs do, so that the data for the two groups are typically fit by the same regression line. Thus, under the assumption that for all observers the same processing steps are recruited by the task and that speed of processing affects all the steps equally (proportionally) the model predicts the same relationship (same slope) between SDs and means at an individual and at a group level.

Our results indicate that the DEM does not adequately fit the data of English readers (Myerson et al., 2003). Notably, this model has been largely developed on experiments run on English samples, although typically not on reading tasks (Myerson et al., 2003). So, the differential outcome may indeed be specific to reading.

## Discussion

Are group means effective estimates of reading speed? Results of Experiments 1 and 2 strongly indicate that the reliable difference between the two language groups is expressed by the variability in the distribution of performances rather than by their mean. Studies with the RSVP reading speed in English participants have been unable to clearly ascertain a value for reading speed, producing speeds that differ in wpm up to a factor of 5 (Rubin and Turano, 1992; Latham and Whitaker, 1996; Fine et al., 1997, 1999; Chung et al., 1998; Pelli et al., 2007). Differences across studies may be partially explained by the diverse procedures adopted: presence, or absence, of a mask preceding and following the stream of words in the trial, presence, or absence, of context (i.e., random words vs. sentences), number of words presented in a trial (for an overview on the effects of these variables in RSVP reading see Primativo et al., in preparation). However, our results indicate that part of the differences in reading speed estimates obtained by different laboratories may be related to sampling biases. English readers are much more variable; thus, sample size and selection criteria greatly affect the reliability of the mean in defining the group speed (especially for the small sample sizes typical of these studies). Our results indicate that differences in speed across labs may be in part reconciled in light of the large variability shown by the English population.

Also results with vRTs (Experiment 3) point to the presence of greater differences in variability between English and Italian observers than in terms of mean performances. However, results in this case indicate that different parameters capture the group differences in variability: English observers were more variable for σ and τ values but not for μ, i.e., the mean of the Gaussian component.

How can these differences be accommodated? One possible interpretation is that, by minimizing the role of memory, pronunciation time and eye movements, performance in the RSVP paradigm closely captures the efficiency in decoding; so, individual differences are directly reflected in differential ranges, with the English sample containing both the slowest and fastest individuals. In the case of RT measures, the available literature indicates a more complex relationship between performance and decoding. There is a consensus that RT measures contain both decisional and non-decisional components although there are different approaches to separate them (e.g., diffusion model: Ratcliff et al., 2004; DEM: Myerson et al., 2003). Within the DEM to which we refer here, RTs are a compound of a sensory-motor compartment and of a decisional compartment. Myerson et al. (2003) propose that individual differences are confined to the decisional component of the response. In this perspective, it is not surprising that individual differences are not well captured by variations in the mean as this expresses both decisional and non-decisional components of the response. To provide a general frame for this distinction consider that, based on DEM, in the present experimental conditions the sensory-motor and cognitive compartments each account for about half of the processing time in the Italian sample. Thus, 239 ms was the estimate for the former compartment; subtracting this value from the overall mean (502 ms), we obtain an estimate of 263 ms for the cognitive compartment. Note that the two compartments were not distinguishable among English observers (see further comments below).

Furthermore, in keeping with the idea that the typical skew to the right of RTs increases with test difficulty (Wagenmakers and Brown, 2007), we observed that τ values captured changes in performance as a function of the lexical status of the stimulus better than μ values. This pattern is consistent with previous observations on both reading (Yap et al., 2012) and non reading (Schmiedek et al., 2007; Tse et al., 2010) tasks. Accordingly, we found that English observers were more variable in their τ values. So, within this reasoning, the outcome of the three experiments can be reconciled by stating that English observers showed greater individual differences than Italian observers in the parameter which, in each paradigm, is sensitive to variations in task difficulty (lexical status in our case), i.e., mean values in the case of the RSVP and τ values in the case of the vRTs. Note that in the case of vRTs, English observers were more variable than Italian observers also in terms of the variability of the gaussian component of the response (σ). Further comments will be made on this point when commenting the results within the DEM model.

Is there a processing speed difference in reading in regular and irregular orthographies once (most) cognitive variables are taken into account to match stimuli across languages? Experiment 2, but not Experiments 1 and 3, showed that English readers were faster than Italians. However, in all three experiments the English observers were more variable (although on different critical parameters). English and Italian differ in the degree of consistency in the mappings of letters onto sounds as well as in the complexity of the syllabic structure. The lower syllabic complexity of Italian language enables for easy segmentation of words into phonemes/syllables and, in turn, to effectively acquire grapheme-to-phoneme mappings. On the other hand, in English, the embedding of grapheme-phoneme correspondences in consonant clusters makes it more difficult to acquire these correspondences. In fact, Seymour et al. (2003) found that syllabic complexity affects accuracy and speed of reading non-words (although not familiar words) and exaggerates the lexicality effect. Moreover, it has been suggested that the preferred grain size unit (i.e., the number of graphemes and phonemes) of the lexical entries differ across languages and determines different developmental constraints as well as the characteristics of adult fluent reading (Ziegler and Goswami, 2005). Ziegler et al. (2001) compared word and pseudo-word reading of German (a regular orthography) and English participants reading identical words (words written identically in the two languages such as ball, park, and hand) as a function of their length. Results showed that reading 5- and 6- letter words, the German participants were about 50 ms slower than the English sample. Conversely, Paulesu et al. (2000) found that adult Italian readers were faster at recognizing both words and pseudo-words relative to English readers. Frith et al. (1998) measured reading accuracy and speed of German and English children ranging in age between 7 and 9 years. Interestingly, they found that on average English children read at a slower speed and less accurately than German children, also showing a larger lexical effect. However, selecting a subgroup of “good readers” that made no errors in the easy items, they found English children to be slightly faster than their German peers. The results of the present experiments strongly indicate that English readers are more variable, and that the group mean *per se* fails to capture the phenomenon of the differences across languages. Thus, it is possible that individuals read a language with opaque orthography and complex syllabic structure adopting different processing strategies each contributing to reading with differential efficiency.

One of the aims of this study was to investigate the relationship between the general speed factor and the efficiency of the orthographic decoding on vRTs. Indeed, larger effects of the experimental manipulations are expected in the case of differences in overall processing time across individuals (i.e., larger effects for slower individuals, Faust et al., 1999). In the absence of a general speed difference in vRTs (Experiment 3) no over-additive group interactions are expected. Nonetheless, some data obtained by our research group on English and Italian children in reading single words and pseudo-words may be relevant on this issue (Marinelli et al., submitted). We found that, contrary to the prediction of a larger RT variability in slower individuals (Faust et al., 1999; Myerson et al., 2003), the English sample was generally faster but more variable than the Italian sample across conditions, providing additional evidence that increased variability is a specific characteristic of English readers. Large inter-individual differences have also been found in other studies with English children, both when the English readers were faster (Ellis and Hooper, 2001; Ellis et al., 2004) or slower (Patel et al., 2004) than readers of regular orthographies. Taken together the results of the three experiments show that large variability is not associated with slower speed in the case of the English sample. This highlights the importance of examining the shape of the distributions to understand the underlying phenomenon.

Do the individual differences across languages arise from different strategies adopted in reading? One source of individual difference could arise from readers emphasizing different strategies or types of information during reading. Yap et al. (2012) linked the distributional characteristics with the dynamics of information accumulation over time. They found that a fluent lexical process, measured by good vocabulary knowledge, was associated with more efficient accumulation of information and lower τ. Accordingly, we found that only τ, and not σ or μ, were modulated by the lexical status of the stimuli (i.e., word frequency). If small τ is associated with higher use of the lexical strategy of reading (Yap et al., 2012), this may be more pronounced in the English population (i.e., lower τ indicates a more efficient process). However, word frequency modulated the τ parameter in a similar way in both the English and Italian samples. Again, the insensitivity in detecting mean group differences may be linked to the presence of large individual differences; so, apart from showing lower τ values, English observers were also significantly more variable in this parameter. Therefore, we feel that the possibility that the lexical strategy of reading is more pronounced in the English may require further testing before such hypothesis can be confidently rejected.

The DEM assigns the difference between individuals to the amount of cognitive processing required by the task predicting the relationship between mean and SD (Myerson et al., 2003). Applying the DEM to the data of Experiment 3 revealed that, in the case of the English sample, the SDs were not linearly related to the mean vRTs. There is a large body of literature that builds on the relationship between mean RTs and SDs to account for individual differences across slow and fast groups (e.g., Bashore and Ridderinkhof, 2002; Myerson et al., 2003). These studies investigate different cognitive processes, ranging from recognition to counting (Cerella et al., 1980; Cerella, 1985; Logan, 1992; Mayr and Kliegl, 1993; Hale and Jansen, 1994; Zheng et al., 2000; Palmer et al., 2011). The relationship between the standard deviation and the mean of the RT distribution highlights a general rule (Wagenmakers and Brown, 2007) that must be taken into account when looking for selective effects (Hale and Jansen, 1994; Faust et al., 1999; Zheng et al., 2000; Myerson et al., 2003). Indeed, most models of reading are based on the selective effects of lexical variables (e.g., CDP++ by Perry et al., 2010). Our results indicate that this relationship does not hold for reading speed in English. Note that, in a counting task, English participants show the expected relationship (Logan, 1992) but, as shown here, this is not the case for reading. Therefore, this makes a special case for English individuals and the reading task.

It is difficult to understand why the general linear law between means and standard deviations does not hold in this particular instance. Wagenmakers and Brown (2007) identify three boundary conditions under which no linear relationship between means and standard deviations is expected, i.e.: (a) manipulations affecting non-decision times; (b) mixtures (i.e., two different decisional processes going on at the same time) and (c) serial and exhaustive processing. An example of a mixture is when a task (e.g., counting dots) is solved in a moment of transition between the use of an algorithm (typically used in the early stages of learning) and of an automatic retrieval strategy (as in the case of the instance theory; Logan, 1992). Reading models can be seen in this perspective. Thus, at least to some extent, the dual route model (Coltheart et al., 2001) appears compatible with the instance theory, in that the sub-lexical route relies on an algorithm and the lexical route activates individual traces from the orthographic lexicon (i.e., specific instances). However, it seems extremely unlikely that English proficient adults are in a moment of transition between the two routes (if anything, this interpretation could apply more easily to Italian readers who supposedly develop their lexicon more slowly). The third boundary condition also does not seem to apply to the present data; it seems unlikely that reading is carried out through serial and exhaustive processing. At any rate, were this the case, one would expect means to vary linearly as a function of variances, rather than SDs (Wagenmakers and Brown, 2007). However, the results for English readers shown in Figure 4 remained the same when we used variances instead of standard deviations, suggesting, as expected, that failure for linearity between means and SDs is not related to the task involving a serial and exhaustive processing.

Evaluating the first boundary condition (i.e., manipulations affecting non-decision times) identified by Wagenmakers and Brown (2007) is more complex. In general, models do not predict a relationship between means and standard deviations for the non-decisional component of the response. This is the case for the diffusion model (Ratcliff, 1979, 2002) as well as for the DEM (Myerson et al., 2003) to which we refer here. Variations among individuals and tasks certainly cannot be simply viewed as due to differences in sensory processing and motor preparation processes. However, in evaluating this boundary condition, it should also be considered that any systematic bias in the modulation of the response (such as response conservativeness) would be incompatible with the linear law (Wagenmakers et al., 2005). In this perspective one may consider the time criterion account for naming speed advanced by Lupker and colleagues in a number of studies carried out on English speaking individuals (e.g., Lupker et al., 1997; Taylor and Lupker, 2001; Kinoshita and Lupker, 2002; Chateau and Lupker, 2003). According to this hypothesis, participants set a point in time at which they try to respond to all stimuli in a given block. When easy and hard stimuli are mixed, the placement of the time criterion is intermediate compared with that in pure blocks of easy and hard stimuli; thus, responses to easy stimuli slow down and responses to hard stimuli speed up (thus altering the relationship between speed of response and task difficulty which is at the base of the linear law). Notably, recent evidence (Paizi et al., 2010) indicates that a time criterion account does not easily explain reading RTs in Italian young adults. Thus, unlike what is typically reported in English-speaking individuals, word frequency effects are independent of list context manipulations (Paizi et al., 2010). So, one possibility is that the atypical pattern in the relationship between means and standard deviations is due to the fact that English readers (more so than readers of a regular orthography) refer to a time criterion when they try to read under speeded time conditions.

This hypothesis may also be instrumental in understanding the difference in intra-individual variability observed between the two linguistic groups: English observers present much higher σ values. Presumably, these values in part reflect the degree of noise in the data (whether arising from decisional or non-decisional components of the response). In this vein, it is interesting to note that σ values are extremely small in Italian observers, averaging 35.7 ms, with also very little inter-individual variation (*SD* = 18.7). If we assume that only English observers superimpose a time criterion on their response it becomes reasonable to imagine an increase in their individual intra-trial variability (independent of mean changes). Indeed, adopting a time criterion modifies the pattern of individual response in a way which, on the one hand, does not appreciably modify mean performance and, on the other, is inherently symmetrical and, as such, at least compatible with a Gaussian distribution. As the time criterion reflects a selective bias in the response, this perspective would also help understanding why σ values are insensitive to the lexical properties of the stimulus. Overall, we propose that the increase in intra-individual variability shown by English observers might be interpreted as due to a combination of two factors, intra-trial noise and reference to a time criterion for setting up the response. Only the former factor would be active in the case of individuals reading a very regular orthography, such as Italian. Clearly, further *ad hoc* research designs are needed to fully evaluate this interpretation.

Most universal models of reading and reading acquisition are based on mean vRTs of English participants as a function of lexical manipulations (e.g., Seidenberg and McClelland, 1989; Coltheart and Rastle, 1994; Coltheart et al., 2001; Perry et al., 2007, 2010). The current results question the appropriateness of building a universal reading model just on the very language group who in reading performance does not conform to the general predictions of models of RT performance.

### Conflict of interest statement

The authors declare that the research was conducted in the absence of any commercial or financial relationships that could be construed as a potential conflict of interest.
