# Supplementary material for: Does the mean adequately represent reading performance? Evidence from a cross-linguistic study
Source: Front Psychol. 2014 Aug 19;5:903. doi: 10.3389/fpsyg.2014.00903 (PMC4137238; doi:10.3389/fpsyg.2014.00903)
Supplement: Supplementary file 1 [file DataSheet1.PDF]

Appendix A

Psycholinguistic characteristics of words included the Italian and English lists used in Experiment 1.

	**Italian list**		**English list**	
	mean	SD	mean	SD
Words frequency	25.06	24.37	24.77	36.19
N-size	1.90	2.02	2.36	1.75
Age of Acquisition	3.78	1.03	3.84	0.94
Imageability	4.91	1.09	5.02	0.85
bigram frequency	10.51	1.43	10.39	0.48
n° of phonemes	4.75	0.41	4.01	0.70
n° of syllables	2.00	0.00	1.29	0.46

Note: Values refers to the LEXVAR database (Barca et al., 2002) and the Colfis database (Bertinetto et al., 2005) for Italian (referring to one million of occurrences), and to the MRC Psycholinguistic Database 2.0 (Wilson, 1988), the CELEX database (Baayen et al., 1993) and the MCWord database (Medler and Binder, 2005) for English list. N-size indicates the mean number of neighbours. Values of imageability and age of acquisition indicate the mean ratings given by university students on a 7-point scale. Bigram frequency values were express as natural logarithm.

Appendix B

Psycholinguistic characteristics of words included in the Italian and English lists used in Experiment 2 (see also notes to appendix A).

	**Phoneme-marched list**				**Letter-marched list**			
	**Italian**		**English**		**Italian**		**English**	
	mean	SD	mean	SD	mean	SD	Mean	SD
Words frequency	27.56	88.44	22.13	24.31	29.12	64.39	24.77	36.19
N-size	1.63	2.20	1.91	1.98	2.42	2.24	2.36	1.75
Age of Acquisition	3.89	1.18	4.04	0.93	3.71	1.31	3.84	0.94
Iimageability	4.97	1.03	4.85	0.86	5.06	1.08	5.02	0.85
bigram frequency	10.79	0.45	10.43	0.40	10.80	0.65	10.39	0.48
N° of letters	5.45	0.69	6.03	0.90	5.00	0.00	5.00	0.00
N° of phonemes	5.00	0.00	5.00	0.00	4.15	0.36	4.01	0.70
n° of syllables	2.00	0.00	1.76	0.43	2.00	0.00	1.29	0.46

Appendix C

Psycholinguistic characteristics of words included in the Italian and English lists used in Experiment 3 (see also notes to appendix A).

	**Phoneme-marched list**								**Letter-marched list**							
	**high frequency words**				**low frequency words**				**high frequency words**				**low frequency words**			
	**Italian**		**English**		**Italian**		**English**		**Italian**		**English**		**Italian**		**English**	
	mean	SD	mean	SD	mean	SD	Mean	SD	mean	SD	mean	SD	mean	SD	mean	SD
Words frequency	55.26	78.51	63.54	14.49	2.56	1.24	2.86	1.18	56.88	56.68	61.29	18.80	2.66	1.33	2.85	1.92
N-size	1.26	1.88	1.47	1.19	2.50	2.75	1.67	1.29	2.71	2.62	2.40	1.45	2.08	2.27	1.67	1.54
Age of Acquisition	3.44	0.94	3.71	1.00	4.25	1.19	4.72	0.82	2.94	1.10	3.56	0.87	4.00	0.95	4.41	1.01
Iimageability	5.02	1.22	4.66	1.00	4.73	1.04	4.71	0.66	5.39	0.93	4.69	1.04	5.00	0.94	4.81	0.81
bigram frequency	9.85	1.28	10.46	0.40	10.34	1.11	10.51	0.36	10.15	0.92	10.28	0.44	10.01	1.48	10.24	0.50
N° of letters	6.00	0.82	6.00	0.85	5.70	0.69	6.33	1.05	5.00	0.00	5.00	0.00	5.00	0.00	5.00	0.00
N° of phonemes	5.00	0.00	5.00	0.00	5.00	0.50	5.00	0.00	4.05	0.22	4.27	0.80	4.25	0.44	4.00	0.76
n° of syllables	2.00	0.00	1.80	0.41	2.00	0.00	1.80	0.41	2.00	0.00	1.27	0.46	2.00	0.00	1.40	0.51

Appendix D

Mean individual vRTs calculated over items and relative SDs for the Italian and English sub-group. The table also reports the three parameters values resulting from the ex-Gaussian fits (μ: mean of the Gaussian distribution; σ: standard deviation of the Gaussian distribution and τ: mean of the exponential component).

Subject ID	Italian participants					English participants				
	Mean	SD	μ	σ	τ	Mean	SD	μ	σ	τ
1	529	118	432	53	97	444	77	442	77	2
2	680	115	563	33	116	399	72	402	56	2
3	557	126	468	14	96	500	104	394	37	107
4	533	98	468	69	65	494	168	516	126	4
5	470	87	399	45	70	523	107	456	45	75
6	510	87	442	24	68	456	56	417	38	39
7	477	82	409	25	68	485	91	431	72	54
8	576	97	484	20	92	537	167	501	149	43
9	572	98	490	34	82	514	104	525	64	2
10	486	104	395	34	90	326	102	342	70	2
11	518	101	443	55	74	564	94	562	93	3
12	495	84	414	23	81	538	113	456	64	82
13	552	86	465	2	86	469	148	420	67	74
14	403	37	369	17	34	457	86	402	60	56
15	457	64	400	22	57	548	126	462	42	93
16	425	56	393	43	32	475	89	435	44	46
17	456	78	384	23	72	486	86	422	54	64
18	477	102	390	26	86	504	157	451	119	65
19	470	96	387	43	84	465	77	463	76	2
20	429	43	406	36	23	475	60	442	49	33
21	497	61	444	25	53	420	150	315	108	105
22	521	90	458	59	63	432	125	440	104	3
23	578	102	511	74	67	411	116	413	106	3
24	444	68	443	67	2	489	223	411	140	117
25	596	88	518	39	78	479	63	478	63	2
26	532	81	467	30	66	595	260	588	50	102
27	437	43	396	17	41					
28	542	117	434	25	108					
29	442	40	407	21	35					
30	500	100	407	32	93					
31	537	66	484	35	53					
32	479	81	477	80	2					
